# Comparative study of optical coherence tomograph and histological images of eustachian tube nasopharyngeal region and adjacent structures in vivo and ex-vivo miniature pigs

**DOI:** 10.1186/s12938-023-01104-z

**Published:** 2023-05-13

**Authors:** Xiao-Mei Sun, Zhi-Wen Xiao, Jia-Qi Luo, Qing-Yu Gu, Hui-Qing Zhang, Bai-Ling Li, Shi-Min Zhuang, Guan-Ping Zhang

**Affiliations:** 1 Department of Otolaryngology Head and Neck Surgery, The Sixth Affiliated Hospital, Sun Yat-sen University, Guangzhou, 510655 China; 2 Department of Otolaryngology, Shenzhen City Baoan District Women’s and Children’s Hospital, Baoan District, Shenzhen, 518100 Guangdong China; 3 Shenzhen Science & Technology Development Exchange Center, Shenzhen, 518000 China

**Keywords:** Optical coherence tomograph (OCT), Histological images, Miniature pigs, Eustachian tube, Nasopharynx

## Abstract

**Objectives:**

Optical Coherence Tomograph (OCT) imaging technology can be used to examine, in vivo, the human ET. At present, it is impossible to achieve the OCT scanning vivo and ex vivo in the same individual human body, or study the consistency between OCT images and histological images of the eustachian tube nasopharyngeal region and adjacent structures. The aim of this study was to determine the consistency between OCT images and histological sections in vivo and ex vivo in miniature pigs.

**Methods:**

OCT imaging was performed on five adult miniature pigs in vivo and ex vivo. The images of the eustachian tube OCT (ET-OCT), nasopharynx OCT (NP-OCT) and histological cross sections were further studied.

**Results:**

All five miniature pigs achieved the OCT scan successfully, acquiring ET-OCT and NP-OCT images in vivo and ex vivo on both sides. The acquired ET OCT images closely matched the histological images, revealing details of the cartilage, submucosa, glands, and mucosa. The lower segment of the ET wall mucosa had an abundance of glands and submucosal tissues, with more low-signal areas appearing in the ex vivo images. The NP-OCT images of the nasopharynx matched the details of the mucosa and submucosal tissues. The ex-vivo OCT images showed thicker mucosa and more scattered slightly lower signal areas compared to the vivo OCT images.

**Conclusions:**

ET-OCT images and NP-OCT images matched the histological structure of eustachian tube nasopharyngeal region structures in miniature pigs both in vivo and ex vivo. OCT images may be sensitive to changes in edema and ischemia status. There is a great potential for morphological assessment of inflammation, edema, injure, mucus gland status.

## Background

The eustachian tube, nasopharyngeal region and adjacent structures are located deep in the skull, making biopsy is difficult. Additionally, biopsies of eustachian tube and nasopharynx are invasive procedures that can impair eustachian tube function and cause symptoms such as bleeding, otitis media, aural fullness and more. Particularly, accurate biopsies are difficult to perform when lesions are submucosa.

Optical coherence tomography (OCT) is a new imaging technology which can penetrate tissue surfaces and generate high resolution images. In an ex-vivo sheep study, superficial structures including the respiratory epithelium, submucosa, cartilage, mucous glands, lymphoid follicles and bone could be identified [1]. Our previous study demonstrates that OCT imaging technology can be used to examine the eustachian tube via its pharyngeal opening in humans, obtaining high-resolution OCT images [2]. It can delineate the morphology of the lumen, mucosa and submucosa. However, currently, it is not possible to achieve the OCT scanning in vivo and ex-vivo in the same individual human body to evaluate the consistency between OCT and histological images of the eustachian tube nasopharyngeal region and adjacent structures. Matching the OCT image features obtained in laboratory animals with their histological features can provide strong evidence for relationships in OCT images from humans. Therefore, it is necessary to conduct a corresponding validation analysis in an appropriate animal model.

In this study, we used the miniature pig, a large experimental animal, to simulate OCT imaging procedures in humans. The congruent relationship between OCT images and histological cross-sections was analyzed.

## Results

### OCT probe implantation

**Fig. 1:**
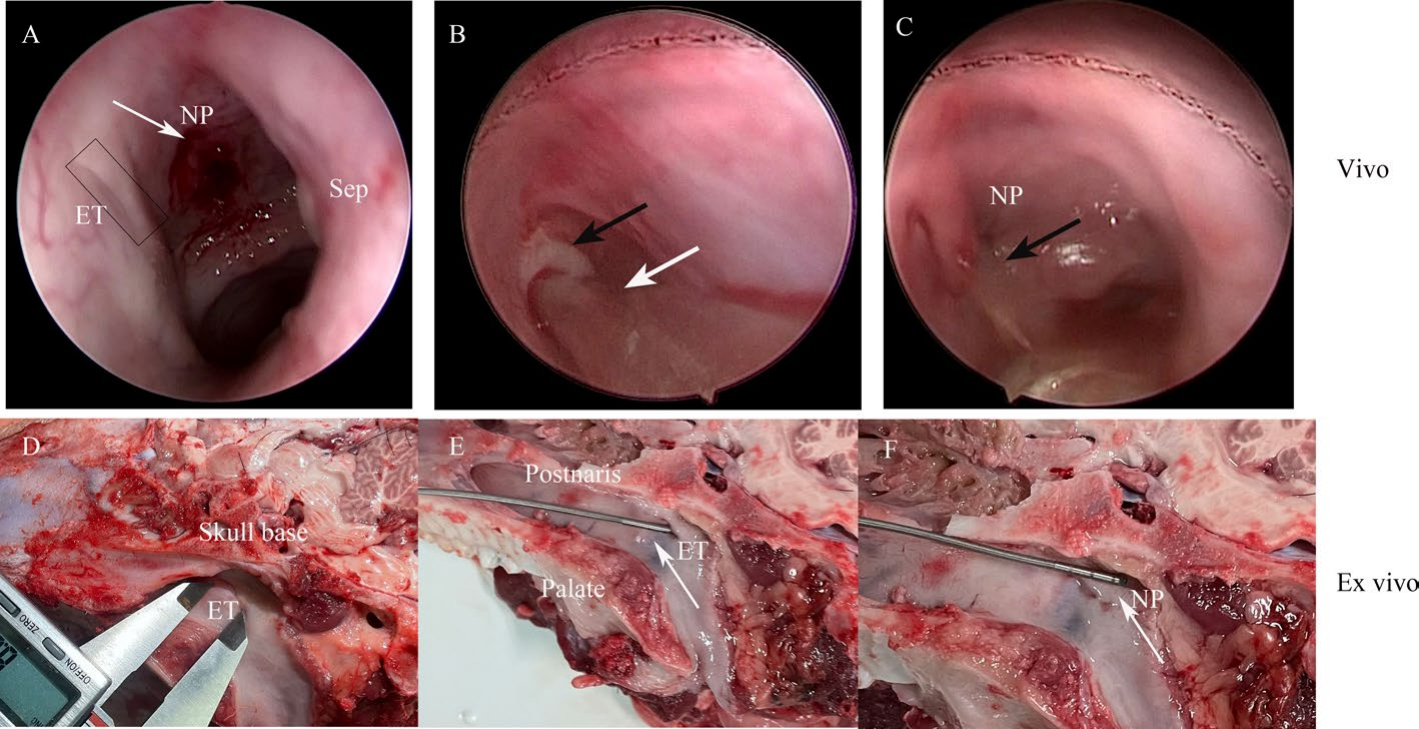
Procedure of ET-OCT and NP-OCT scanning in vivo and ex vivo miniature pigs (right side). **A**, View of the eustachian tube nasopharyngeal region and adjacent structures under nasal endoscopy. Eustachian tube pharyngeal orifice (black rectangle), nasopharynx (). **B**, ET-OCT probe ( ) was inserted into the eustachian tube () and ET-OCT scanning was performed. **C**, NP-OCT image acquisition in progress. **D**, the skull was split in half along the nasal septum, exposing the pharyngeal opening of ET, nasopharynx, pharyngeal recess, postnaris, skull base, palate. **E**, ET-OCT image acquisition ex vivo. Eustachian tube ( ). **F**, NP-OCT image acquisition ex vivo. Nasopharynx ()

In all five miniature pigs, clear views of the nasal structure, pharyngeal orifice of ETs and nasopharynx were obtained. The OCT probe was successfully inserted into the ET lumen and nasopharynx on both sides with the guidance of nasal endoscopy. Both ET-OCT and NP-OCT imaging were performed successfully (Fig. 1).

### Histomorphological findings of miniature pig eustachian tube

**Fig. 2:**
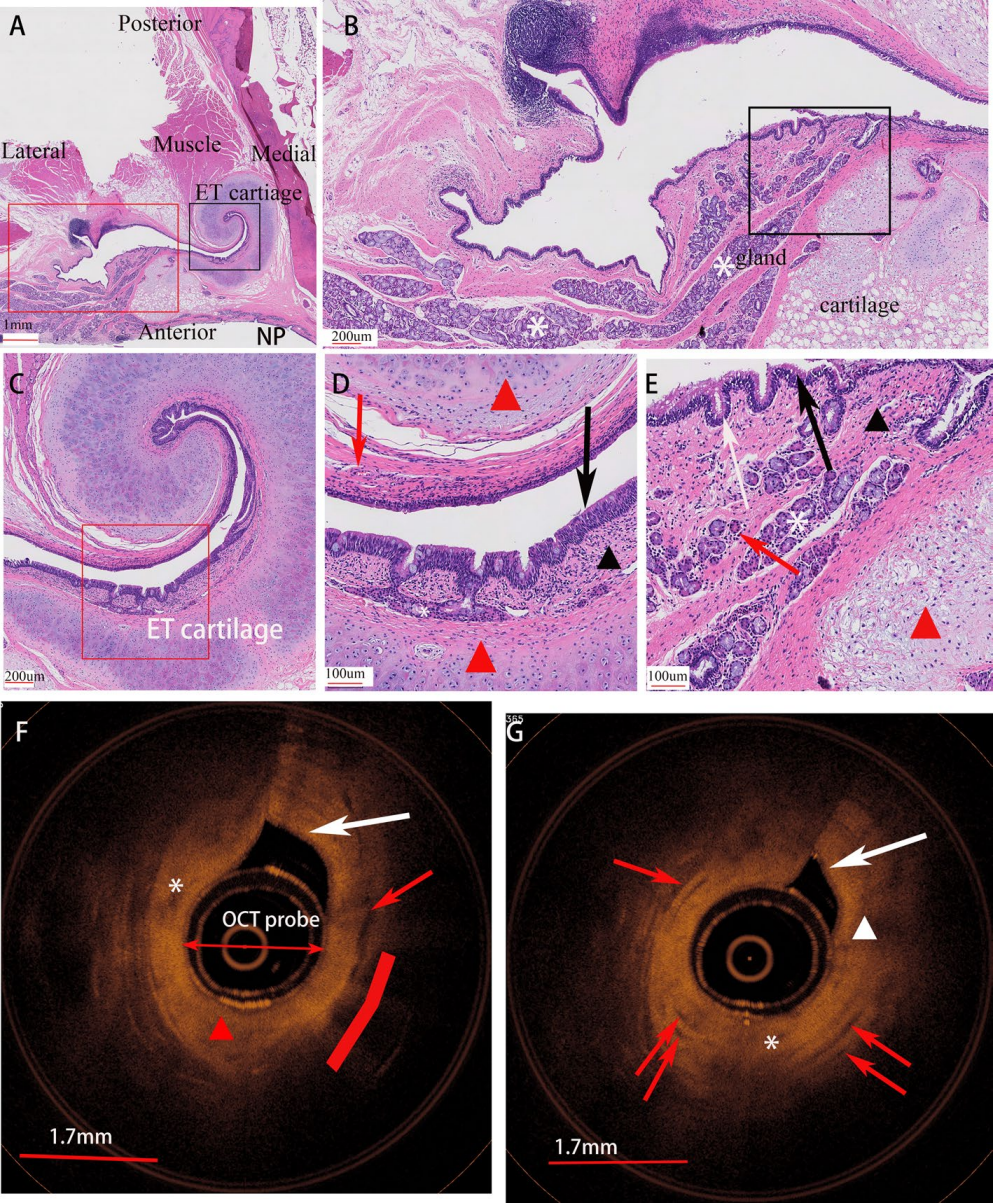
Histological morphology of eustachian tube of miniature pig (right ET, axial). **A**, Whole view of eustachian tube and surrounding tissue. **B**, view of the gland (asterisk) rich area of ET lower segment lumen (4 × power field views of red box in A). **C**, view of the cartilaginous ET from the ET pharyngeal opening (4 × power field view of black box in A). **D**, Higher magnification micrograph of the tubal microstructure (red square in C) shows epithelium (black arrow) with crypt, thin connective tissue (red arrow) between the cartilage (red triangle) and epithelium, a few gland (asterisk), vessel (red arrow) and in the mesenchymal tissue (black triangle) underlying the epithelium. **E**, Higher magnification micrograph of the tubal microstructure (black square in B), shows epithelium (black arrow), crypt (white arrow), thick connective tissue (black triangle), vessel (red arrow) between the cartilage (red triangle) and epithelium, abound gland (asterisk) and mesenchymal tissue underlying the epithelium. ET-OCT image from the ET pharyngeal opening (**F**) and ET lower segment lumen (**G**), shows cartilage (red arc line), epithelium (white arrow), connective tissue (white triangle), gland (red arrow), vessel (white asterisk)

The ET lumen is lined with ciliated pseudostratified columnar epithelium (Fig. 2D, E). Histological manifestations of the ET pharyngeal opening and ET lower segment were different in a certain extent. The ET wall at the nasopharyngeal end consisted the cuboidal cells and thin submucosa. A thin layer of submucosa can be observed between the cartilage and the epithelium (Fig. 2C, D). Connective tissue such as collagen fibers and a few blood vessels can be found in the submucosa. The ET wall at the lower half of the ET lumen consisted of mucosa with secretory mucous cells, columnar cells and abundant submucosa glands. (Fig. 2B, E). The submucosa in the ET lower segment is thicker than that in the ET pharyngeal opening, with more glands and connective tissue. The ET-OCT image from the ET pharyngeal opening shows a thinner connective tissue layer compared to the ET-OCT image from the ET lower segment. In particular, more poorly reflective tissue is visible, which is consistent with glandular tissue in the submucosa.

### ET-OCT images from the pharyngeal orifice and lower lumen of ETs

**Fig. 3:**
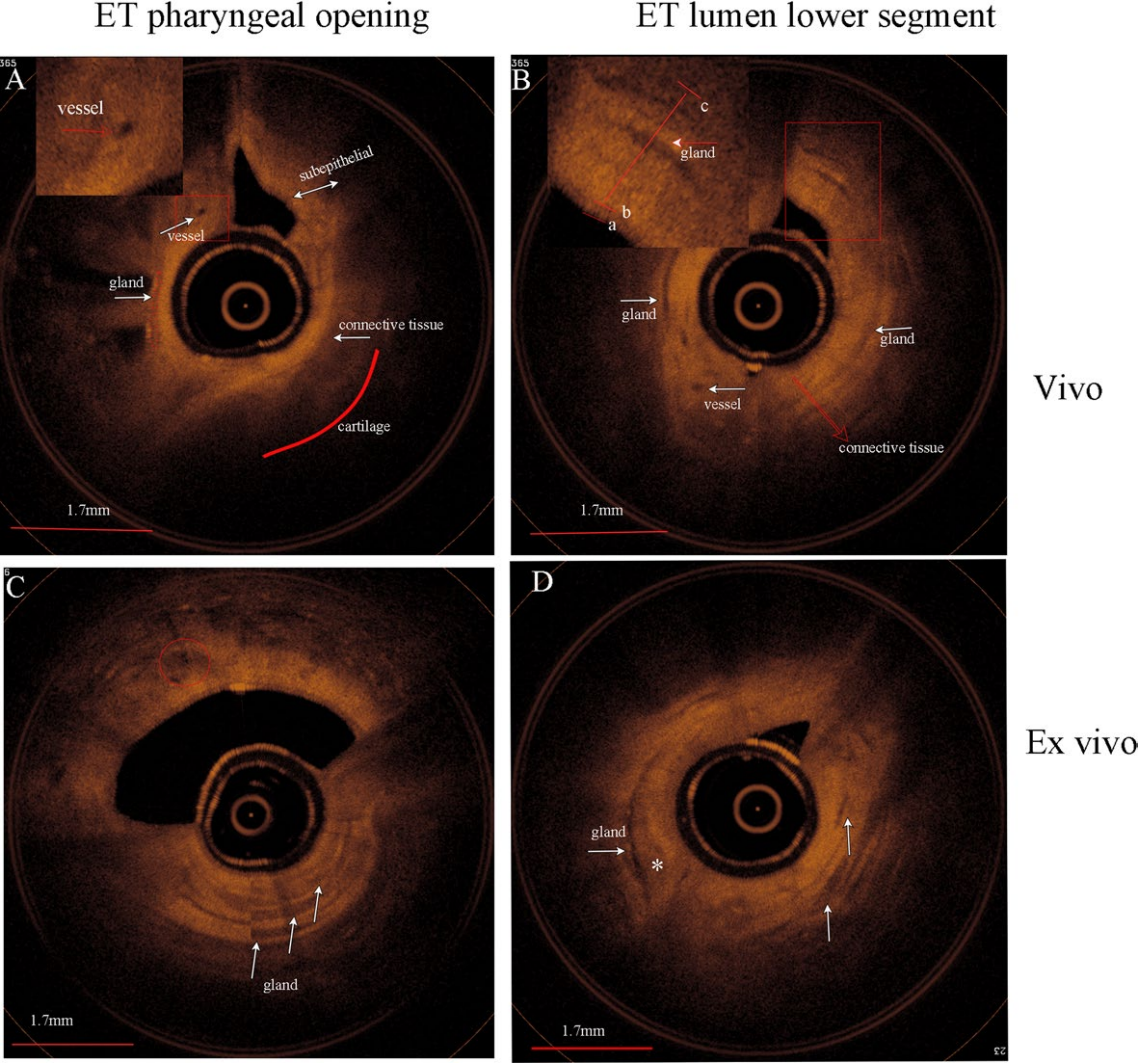
ET-OCT images of eustachian tube in vivo and ex vivo (right ET). Image obtained from ET pharyngeal opening (**A**) and ET lumen lower segment. (**B**) in vivo. **A**, vessel demonstrates a circular signal-poor region (inset, arrow). **B**. Epithelium and submucosa can be shown (inset). Image obtained from ET pharyngeal opening. (**C**) and ET lumen lower segment. (**D**) in ex vivo. **C**, multiple glands with low signal areas (white arrow) and a slightly low signal area (circle) are seen in the submucosa. **D**, larger gland with low signal areas (white arrow) and slightly low signal area (asterisk) are seen in the submucosa. Cartilage (red arc), ab, epithelia, bc, subepithelial

High-quality ET-OCT images were acquired in ten eustachian tube of five miniature pigs both in vivo and ex-vivo, the microarchitecture was illustrated in Fig. 3. The ET-OCT images presented a clear distinct picture of ET lumen, epithelium, submucosa, glands and cartilage of ET wall. On the ET-OCT images, the cartilage appears as a homogeneous low signal area, adjacent to the submucosa. The mucosal layer lined the eustachian tube lumen surface and presented as a slightly lower signal area facing the eustachian tube lumen. It was thin and linear, sometimes indistinguishable from the submucosa and probe. The submucosa showed a slightly higher signal area. Due to the variation of gland and blood vessel components in submucosa, scattered layers of low signal areas are included in it.

The mucosa at the pharyngeal opening of ET is thinner than the lower segment ET. The lower segment ET wall mucosa is abundant of glands and submucosal tissues. This feature can be observed in the ET-OCT images from both in vivo and ex-vivo. (Fig. 3B, D). Meanwhile, the ex-vivo ET-OCT images showed thicker mucosa at the pharyngeal opening of ET, compared to the in vivo ET-OCT images. Sightly low signal areas emerged in the ex-vivo ET-OCT images. Moreover, more low-signal areas and some slightly low signal areas appeared on the ET lumen lower segment ET-OCT images in ex-vivo. It may suggest that tissue edema and ischemia occurred after death, which resulted in submucosal tissue edema and projected corresponding changes on ET-OCT images.

**Fig. 4:**
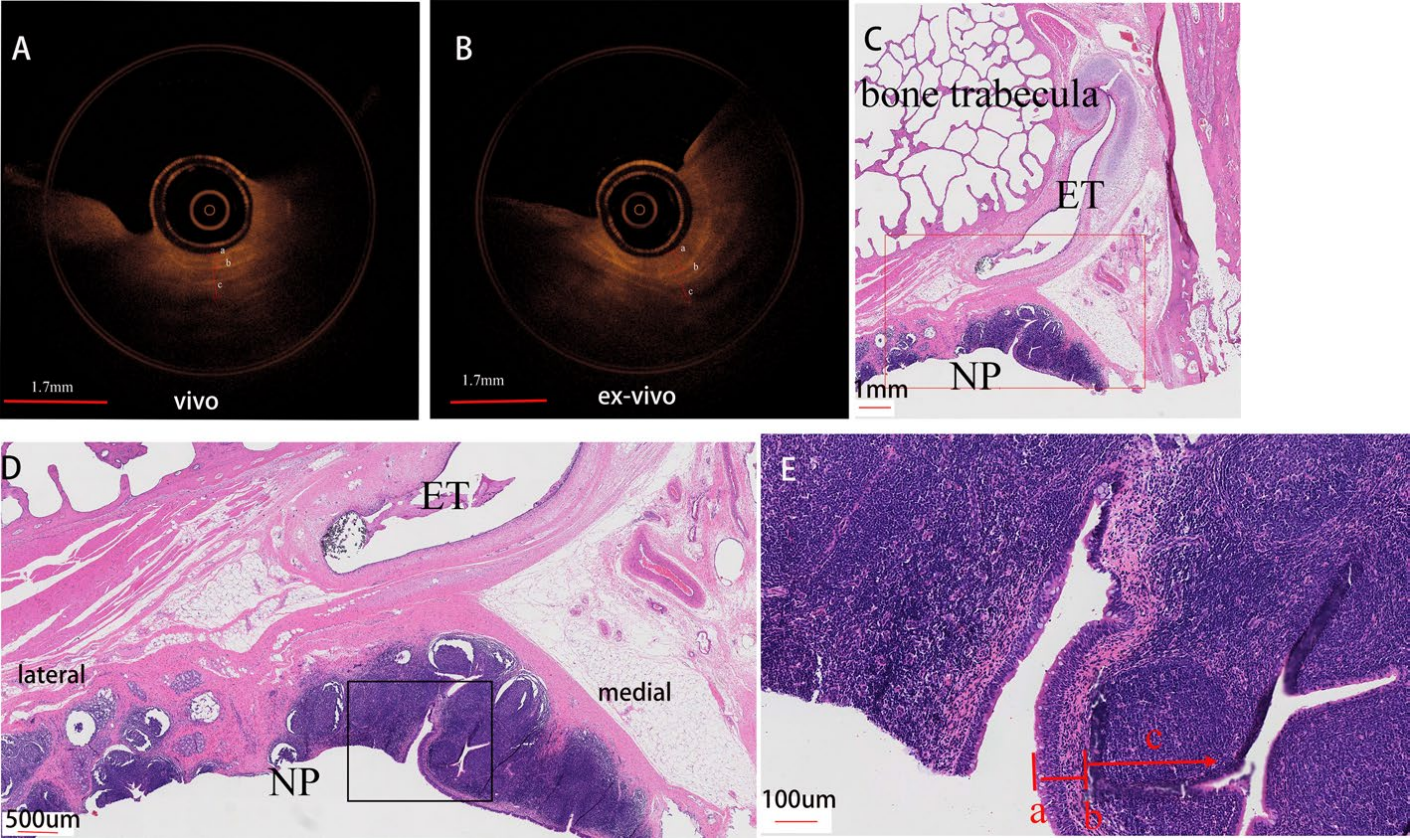
NP-OCT and histological images of the posterior wall of the nasopharynx in vivo (**A**) and ex vivo (**B**) miniature pig (right, axis). OCT image shows the epithelium (a), basement membrane (b), submucosa (c). **C**, Histological view of posterior wall of the nasopharynx and the adjacent structure (0.75x). **D**, Higher magnification view of the red box in **C** (2x). **E**, Higher magnification view of the black box in **D** (10x). Histological image presents the epithelium (a), basement membrane (b), submucosa (c)

**Fig. 5:**
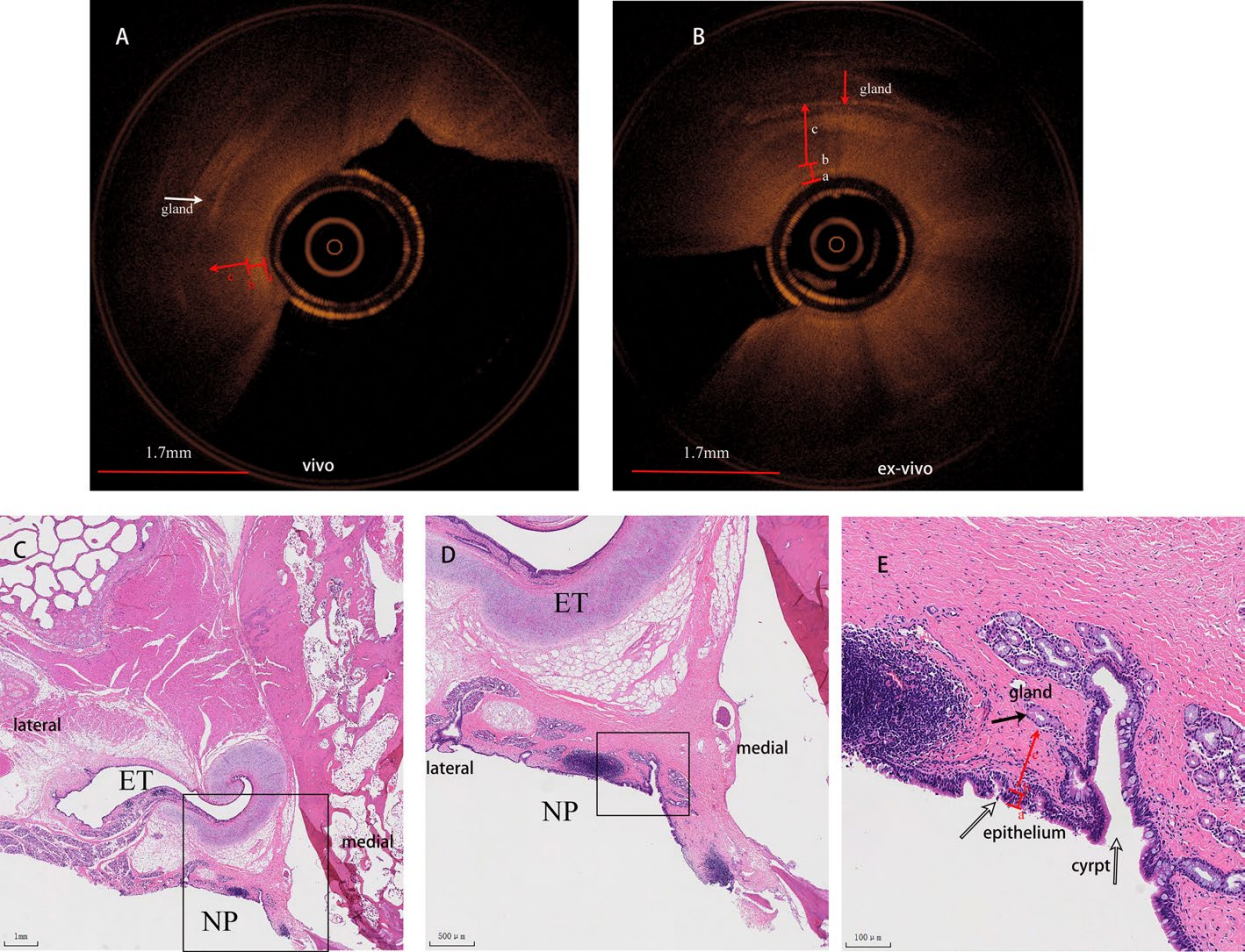
NP-OCT and histologic images of pharyngeal recess in vivo (**A**) and ex vivo (ex-vivo) (**B**) miniature pig (right, axis). OCT image presents the gland (white arrow), epithelium (a), basement membrane (b), submucosa (c). **C**, Histological view of the pharyngeal recess and the adjacent structure (0.74x). **D**, Higher magnification view of the view of the black box in **C** (2x). **E**, Higher magnification view of the black box in **D** (10x). histological image shows the crypt (white arrow), gland (black arrow), epithelium (a), basement membrane (b), submucosa (c)

The NP-OCT images presented clear distinct picture of mucosa, submucosa of the posterior wall of the nasopharynx. (Figs. 4, 5). On the NP-OCT images, the mucosa layer appears as a low signal area, which is distinguished from the lamina propria with a high-signal zone between the submucosa. These features are consistent with the histological structure of the nasopharynx of miniature pigs.

## Discussion

In our study, we performed OCT to visualize the eustachian tube nasopharyngeal region and adjacent structures in miniature pig model. It can display the surface mucosa, surrounding superficial tissue and cartilage of eustachian tube, the mucosa and submucosa of the nasopharynx at near-histological level in vivo and ex-vivo. Meanwhile, ET-OCT images and NP-OCT images can be displayed and saved in real time. Given the difficulty of a eustachian tube biopsy, it is not possible to obtain OCT images and large histological structures of eustachian tube and its adjacent structures simultaneously in the same human body. However, the use of a large animal model such as the miniature pig can help us to better understand the correlation between OCT images and histology. To our knowledge, this is the first report of nasal endoscopy-guided OCT scanning during nasal endoscopy in vivo animal model.

The principle of OCT has been elucidated in detail in previous studies. The technological basis of low coherence interferometry and optical coherence reflectometer theory and technology, integrates optics, supersensitive detection and computer image processing [3–5]. Water and cells in tissues can affect the absorption, reflection and penetration of light [5]. Different tissue structures, with different cell numbers and water composition, yield different absorption and reflectivity of the OCT light, and the intensity of the signal of OCT images reveals the boundaries of those structures. Low-cellularity tissues such as fat, muscle, cartilage, and bone showed lower reflection [6, 7]. Byun et al. [8]showed that muscle and fat tissue exhibited a low reflection signal on the OCT images, while mucosal lining and connective tissues with high refection single, nasopharyngeal lymphoid tissues presented as multiple spots of high refection in a low-refection background. An ex-vivo animal study of ET also revealed respiratory epithelium is characterized by a definite high intensity reflection [1]. Based on our vivo and ex vivo animal study, ET-OCT can identify the mucosa, submucosa glands, blood vessels and cartilage in real time, owing to different signals reflecting the boundary of ET lumen surface structure and cartilage.

Previous studies also demonstrated that the pseudostratified columnar epithelium, basement membrane, lamina propria with blood vessels, glands, and cartilaginous structures can be visualized in the OCT images from the nasal mucosa [9–13]. Additionally, the thickness of the nasal mucosa and epithelial layer in OCT images of patients with cystic fibrosis increased [9]. Meanwhile, post decongestant images showed increased tissue density and a lack of glandular elements [10]. In our study, both ET-OCT and NP-OCT signal features were consistent with those in previous studies, which is in agreement with histopathological features [1, 8–14] (Figs. 2, 4, 5).

To date, many methods, such as high-resolution CT, MR imaging, endoluminal ultrasonography [15] have been used to evaluate morphology, with varying degrees of success. For the pathological changes in the eustachian tube nasopharyngeal region and adjacent structures, especially the submucosal lesions, repeated CT/MRI examination has the disadvantages of high economic cost, radiation and low tissue resolution. Novel contact endoscopy represented good tissue resolution of 10 um, but low tissue penetration of 100 um. None of the technologies provided significant histological information. Biopsy histological examination can provide a good understanding of the histological structure of ET wall and nasopharynx. Repeated nasopharyngeal biopsies in non-neoplastic patients are not recommended. Therefore, accurate and noninvasive evaluation of pathologic changes in the ET and nasopharynx is crucial to guide treatment. Optical Coherence Tomography (OCT) provides a promising solution, with higher resolution (2–10 um) and deeper tissue penetration (2–3 mm) than other imaging modalities [13, 16, 17]. In addition, it can obtain noninvasive histologic images in real time, allowing for a more accurate evaluation of these structures.

The ET-OCT images obtained at the level of the eustachian tube and at a lower lumen level clearly demonstrated that the mucosa and submucosa of the eustachian tube were thicker in comparison to those at the Eustachian tube pharyngeal orifice. This finding is consistent with the ET histological section images (Fig. 2). Previous human histological studies suggested that the wall of the eustachian tube in the lower segment was rich in submucosal glands. In our study using miniature pigs, we also identified a significant number of submucosal glands and vascular components in the histological sections. ET-OCT images generated from the inferior ET clearly showed that the mucosa and submucosa were thicker than those obtained from pharyngeal orifices (Fig. 2), which was consistent with the previous histological section images [18]. The miniature pig can serve as a reliable animal model for investigating eustachian tube nasopharyngeal region and adjacent structures.

In our study, we observe another interesting phenomenon. Some scattered areas with slightly lower signal areas emerged in the submucosa on ex-vivo OCT images, which are rarely seen in vivo OCT images. This suggests that these subtle signal changes may be due to early changes in tissue necrosis after postmortem ischemia. We noticed that the low signal area in ex-vivo OCT images increased and thickened compared to in vivo miniature pigs. The submucosal glandular structures are markedly thickened, and mucosal thickness also increased. We believed this signal change is due to postmortem hemodynamic changes resulting in gland swelling, which is reflected in the OCT images. Swollen submucosal glands and collagen were more sensitive and visible on OCT images (Fig. 3). The OCT features of submucosal edema, congestion and blood flow changes can be used as an important auxiliary means for morphological evaluation of the eustachian tube in inflammatory and edema lesions. Therefore, we believe that OCT is highly suitable for early detection of subtle anatomical changes in the ET and nasopharynx. OCT can also be used as an important auxiliary means for morphological evaluation such as eustachian tube in inflammatory, edema, injures, mucus gland status, benefiting the etiology and clinical diagnosis.

The scanning component of the OCT probe is located laterally, requiring rotation of the probe to acquire an image. Additionally, the size of the surface in contact with the tissue affects the OCT image obtained during scanning. The nasopharynx is an open planar structure, resulting in mainly arc-shaped NP-OCT images, while the eustachian tube is a luminal structure, leading to mostly circular-like ET-OCT images.

Nowadays, the Intravascular Optical Coherence Tomography (IVOCT) has been applied for many years and has developed Consensus standards for acquisition, measurement, and reporting [19]. However, the application of optical coherence tomography in the eustachian tube nasopharyngeal region is still in its early stages. In the future, more multi-center human clinical studies will be planed out to promote the standardization of ET-OCT and NP-OCT image acquisition and image recognition.

## Conclusions

The current research compared ET-OCT images and NP-OCT images to matched the histological images. Although the two cross-sectional imaging methods differ in their principles and presentation methods, their hierarchical characteristics are generally consistent. Both in vivo and ex vivo, tissue hierarchies were clearly visible in the histological images. OCT images may be sensitive to changes in edema and ischemia status. There is a great potential for morphological assessment of inflammation, edema, injury, mucus gland status. A comparative study of normal mucosal ET-OCT and NP-OCT images and their respective disease states should be conducted in large sample sizes.

## Materials and methods

### Animals and study design

Five healthy adult miniature pigs (aged 6 months, weight 20–25 kg, 3 females, 2 males) were utilized in this study, obtained from Guangdong Animal Experiment Center. This study was approved by the Experimental Animal Ethics Committee of Guangdong Medical Experimental Animal Center (No B202108-3). OCT imaging was performed under anesthesia and after sacrificed. The OCT images and histological cross-sections were further studied.

### OCT protocol

OCT imaging was performed by a skilled ENT doctor, using the device of OCTICS Imaging System (Guangzhou Wistar Medical Technology Company Limited, Guangzhou, China) before and after sacrifice. A probe with outer diameter of 1.7 mm was used to obtain the eustachian tube wall OCT images under the guidance of a nasoscope (Karl Storz, Germany). The detailed parameter details of OCT equipment and probe are consistent with the details in our previous article [2].

### OCT in vivo miniature pigs

Miniature pigs were anesthetized by intramuscular injection of Serrazine hydrochloride (0.1 ml/kg, Shengda Animal Medicine Co. LTD, Jilin, China, 20201101) in left gluteus maximus, and 3% pentobarbital sodium solution (0.2 ml/kg, Sigma Aldrich (Shanghai) Trading Co, LTD, Shanghai, China, P3761.) in right gluteus maximus. After anesthesia, the miniature pigs were fixed in prone position. Cotton plancets soaked with ephedrine and tetracaine were placed into the nasal cavity to contract the nasal mucosa and achieve surface anesthesia. A 0°rigid nasal endoscope (4 mm in diameter) (Karl Storz, Germany) was used to obtain the image of nasal structure, nasopharynx and the pharyngeal opening of eustachian tube, from the right nose to the left nose. With the nasal endoscopic guidance, an OCT probe was inserted into the right eustachian tube until the probe was blocked, and then the OCT scanning performed from inside to the nasopharynx. OCT scanning was performed sequentially with the probe placed in the pharyngeal recess and nasopharynx. Then, the same procedure was performed to obtain OCT images of the left eustachian tube and nasopharynx (Fig. 1).

### OCT in ex-vivo miniature pigs

Miniature pigs were sacrificed immediately by exsanguinating via the internal carotid artery after the in Vivo OCT imaging, and were dissected from the level of the second cervical spine within 0.5 h. The skull was split in along the nasal septum, exposing the pharyngeal opening of eustachian tube, torus tubarius, nasopharynx, pharyngeal recess, posterior margin of inferior turbinate, etc. The OCT probe was inserted into the eustachian tube directly until it was blocked, and the OCT imaging was performed from inside to the pharyngeal opening of eustachian tube. Then the probe was placed in the pharyngeal recess and nasopharynx in sequence.

### Histological section protocol

In this study, we adopted a protocol of a previous eustachian tube histological sections study in sheep [7]. The arcuate fissure of the posterior nostril was identified as the pharyngeal opening of the eustachian tube. We use a bone saw to separate the eustachian tube and surrounding tissue. And the we trimmed the specimens into a tissue block (2*2*2 cm), which contained the whole eustachian tube Pharyngeal opening, nasopharynx, pharyngeal recess, torus tubarius and tympanic opening of auditory tube. Each specimen was washed in physiological saline solution, and fixed in paraformaldehyde (4%, PH 5.4, Servicebio, China) for 2 weeks, and decalcified in EDTA (Servicebio, China). After decalcification was completed, the specimens were divided into small pieces in the axial or coronal direction and numbered in sequence. Then they were dehydrated and embedded with paraffin wax. Slices with a thickness of 4 um were taken every 50 um. and tissues with corresponding thickness were discarded with a saw microtome (Craftek, ZheJiang, China). Specimens divided in the axial direction were sectioned from bottom to top, and specimens divided in the coronal direction were sectioned from front to back. Slices were stained with hematoxylin–eosin automatically (SAKURA, Japan). Tissue slide micrograph and view were performed with a slide scan imaging system (TEKSQRAY, China).

### Image analysis

ET-OCT images were collected by constant velocity extraction of OCT probes from inside to outside of the ET. And ET-OCT images were acquired 10 frames per second, thirty frames were equivalent to 1 cm^2^. The anatomical position of each OCT image can be calculated based on the total number of images and image serial number collected from the acquisition process. For example, number 1 refers to the distal end of the eustachian tube and number 50 refers to the proximal orifice of the eustachian tube. During the NP-OCT acquisition on pharyngeal recess, probe from up to down. Meanwhile, during NP-OCT acquisition of the nasopharynx, the probe scanned from the lateral to the middle to collect images and numbered the images. The whole HE images were numbered according to the number of histopathological sections and the consecutive sections 50 um apart. Axial HE sections were numbered from bottom to top, and coronal HE sections were numbered from front to back. Estimate the anatomic position by the number of sections. OCT images and HE image around the same anatomical region were further analyzed. All numbering procedures and analysis were accomplished by two experienced researchers.

## Data Availability

No.
